# Study design challenges and strategies in clinical trials for rare diseases: Lessons learned from pantothenate kinase-associated neurodegeneration

**DOI:** 10.3389/fneur.2023.1098454

**Published:** 2023-03-08

**Authors:** Aleksandar Videnovic, Helle C. V. Pfeiffer, Anna Tylki-Szymańska, Elizabeth Berry-Kravis, Fatih Ezgü, Jitendra Ganju, Agnieszka Jurecka, Anthony E. Lang

**Affiliations:** ^1^Department of Neurology, Massachusetts General Hospital and Harvard Medical School, Boston, MA, United States; ^2^Department of Child Neurology, Oslo University Hospital-Rikshospitalet, Oslo, Norway; ^3^Department of Pediatrics, Copenhagen University Hospital Hvidovre, Copenhagen, Denmark; ^4^Department of Pediatrics, Nutrition and Metabolic Diseases, Children's Memorial Health Institute IPCZD, Warsaw, Poland; ^5^Department of Pediatrics, Neurological Sciences, Anatomy and Cell Biology, Rush University Medical Center, Chicago, IL, United States; ^6^Department of Pediatrics, Gazi University Faculty of Medicine, Ankara, Türkiye; ^7^Consultant to BridgeBio, San Francisco, CA, United States; ^8^CoA Therapeutics, Inc., A BridgeBio Company, San Francisco, CA, United States; ^9^Department of Medicine (Neurology), Edmond J. Safra Program in Parkinson's Disease, and the Rossy Progressive Supranuclear Palsy Centre, Toronto Western Hospital, University of Toronto, Toronto, ON, Canada

**Keywords:** ultra-rare disease, orphan disease, clinical trial design, inborn errors of metabolism, movement disorders

## Abstract

Substantial challenges in study design and methodology exist during clinical trial development to examine treatment response in patients with a rare disease, especially those with predominant central nervous system involvement and heterogeneity in clinical manifestations and natural history. Here we discuss crucial decisions which may significantly impact success of the study, including patient selection and recruitment, identification and selection of endpoints, determination of the study duration, consideration of control groups including natural history controls, and selection of appropriate statistical analyses. We review strategies for the successful development of a clinical trial to evaluate treatment of a rare disease with a focus on inborn errors of metabolism (IEMs) that present with movement disorders. The strategies presented using pantothenate kinase-associated neurodegeneration (PKAN) as the rare disease example can be applied to other rare diseases, particularly IEMs with movement disorders (e.g., other neurodegeneration with brain iron accumulation disorders, lysosomal storage disorders). The significant challenges associated with designing a clinical trial in rare disease can sometimes be successfully met through strategic engagement with experts in the rare disease, seeking regulatory and biostatistical guidance, and early involvement of patients and families. In addition to these strategies, we discuss the urgent need for a paradigm shift within the regulatory processes to help accelerate medical product development and bring new innovations and advances to patients with rare neurodegenerative diseases who need them earlier in disease progression and prior to clinical manifestations.

## Introduction

Significant study design and methodology challenges exist during development of a clinical trial to examine treatment response in patients with a rare disease, especially one with predominant central nervous system involvement combined with heterogeneity in clinical manifestations and natural history (1). At least in part, these challenges exist because clinical trials in rare diseases with neurodegeneration have been required to focus on endpoints based on symptoms. By definition, this requires the presence of well-established symptomatic disease, which generally indicates an advanced disease state with exhaustion of the brain's reserve or compensatory functions. At this point in disease progression, the expectations for even the most effective disease modifying agent may be limited to stabilizing the disease and prolonging the time the patient has in this impaired state before further progression as the last neurons degenerate more slowly.

A paradigm shift is needed in clinical trials of rare diseases to focus on treatment before the onset of significant clinical disease, preferably pre-symptomatically, rather than end-stage or mid-stage symptoms. The degree of pre-existing disease in any organ can dictate the potential for reversibility of the disease process. Since the brain cannot regenerate neurons upon reversing the disease process, the optimal time to treat a patient with a known neurodegenerative disease is before they are symptomatic, and even as early as birth, to prevent the loss of the reserve neurons and, thus, prevent the disease from manifesting. In the case of inborn errors of metabolism (IEMs), the disease begins *in utero*, although patients may remain asymptomatic during early months or even years. The initial endpoint of such a treatment plan would be evidence from a biomarker, specifically a Primary Disease Activity Biomarker (PDAB) (2), that allows for selective targeting of the specific underlying disease biology rather than symptoms. The PDAB is directly related to disease pathogenesis or disease course and change in the PDAB indicates the drug, at specific dosing, has altered the course of the disease. Given such evidence, accelerated approval of the drug could be granted based on the PDAB. The accelerated approval would be followed by a long (i.e., several years) post-marketing trial that compares disease onset and progression (or lack of progression) to natural history controls. Through this approach, the rare disease could possibly be prevented, and patients could obtain the best possible outcomes rather than remaining in a prolonged disabled state in which the final stages of neurodegeneration are slightly slowed based on clinical symptom assessments, as is currently done in trials targeting neurodegenerative disorders.

At present such a paradigm shift is generally not possible. Many patients do not present until their disease is well established, and biomarkers that qualify as PDAB indexing the core mechanism of disease may not be available. Given current limitations, the development of a clinical trial program in a rare disease requires several crucial study design and methodology decisions. These decisions, which may significantly impact success of the study, include patient selection and recruitment, identification and selection of endpoints, determination of the study duration, and selection of appropriate statistical analyses (3–5). The ongoing relevance of the needs specific to treatment development in rare diseases is illustrated by differing regulatory guidance and differing characteristics of implemented trial designs for rare disease studies as shown in the various trial registries (e.g., clinicaltrials.gov, EU Clinical Trials Register, the Japan Primary Registries Network, and the European Public Assessment Reports for orphan medicinal products) (4–7).

During clinical trial program development for an investigational drug for patients with a rare disease (Table 1), study design challenges could be addressed through several strategies involving (1) review of the natural history of the rare disease of interest (while noting potential limitations of older natural history sources due to disease variability and changes in environmental factors and standards of care over time); (2) review of clinical development programs in the same and other similar diseases (both non-interventional natural history and registry studies and interventional studies); (3) engagement with physicians and specialists managing patients with the rare disorder (including 1:1 interviews and scientific advisory board meetings), with patient advocacy groups, and with regulatory experts and agencies, including the US Food and Drug Administration (FDA) and the Committee for Medicinal Products for Human Use (CHMP)/European Medicines Agency (EMA); (4) interactions with biostatisticians to guide appropriate study design and analysis approaches, including the possibility of piloting small exploratory studies prior to performing a pivotal trial and/or prospective, observational, non-interventional studies early in the clinical development program; and (5) exploring and defining biomarkers that may be used as surrogates for clinical outcomes.

**Table 1:** Clinical development program potential clinical studies: an example using PKAN.

**Clinical studies**	**Study objectives**	**PKAN patient population**	**Corresponding endpoints**
Natural history studies	• Provide a clinical baseline •Quantify rate and variability of disease progression • Aid in detecting safety concerns • Provide context for efficacy evaluation • Help identify biomarkers or other surrogate measures and determine correlations with disease • Help establish the optimal window of intervention • Provide a control cohort that can be enrolled into a treatment study	• All	Time course of: • Clinically relevant disease manifestations (both acute and chronic) • Biomarkers and potential surrogate endpoints
Early clinical studies (phase 1, phase 1/2)	• Safety, tolerability • Pharmacokinetics • Early efficacy (pharmacodynamics biomarkers if available) • Dose selection	• Patients older than 12 years of age (unless prior safety data exists)	• Pharmacodynamic biomarkers and potential surrogate endpoints if available
Registration study (phase 2/3, phase 3, extension studies)	• Safety, tolerability • Efficacy in the main patient population (e.g., classic PKAN) **and** • Longer-term safety and tolerability • Longer-term efficacy	• Patients with early disease onset (classic PKAN) with dystonia onset before the age of 10 years	• Dystonia scales • Patient-reported outcomes
	• Safety, tolerability • Efficacy in a different/additional patient population	• Patients with later disease onset (atypical PKAN) • Presentation: dystonia onset after 10 years of age	• Speech assessments • Patient-reported outcomes
Additional studies (auxiliary studies, DDI studies, QT study, renal/hepatic impairment study)	• Additional safety parameters	• Healthy volunteers • Renal/hepatic impairment patients	• PK and cardiac safety (QT prolongation)
Late clinical studies (phase IV studies, registries)	• Long-term safety and tolerability • Long-term efficacy	• All	• All clinically relevant disease manifestations • Biomarkers and potential surrogate endpoints if available

DDI, drug-drug interaction; PKAN, pantothenate kinase-associated neurodegeneration.

Here we review strategies for the successful development of a clinical trial to evaluate treatment of a rare disease with a focus on IEMs that present with movement disorders. We focus on registrational study designs as they pose the greatest design challenges. The strategies are presented using pantothenate kinase-associated neurodegeneration (PKAN) as the rare disease example with consideration given to other IEMs with movement disorders. Lessons learned from prior PKAN studies as well as other rare neurodegenerative genetic diseases are discussed and the application of the strategies to other rare diseases with neurodegeneration and movement disorders (e.g., other neurodegeneration with brain iron accumulation [NBIA] disorders, lysosomal storage disorders, and other IEMs) is presented.

IEMs are defined as any condition that leads to a disturbance of a defined metabolic pathway and include not only enzyme or transporter deficiencies or superactivities, but also chaperone and transcription factor abnormalities (8). They encompass a large group of single gene disorders that can affect all organs and lead to a variety of symptoms (9). Movement disorders are among the most common neurological symptoms in children with IEMs and account for a significant part of the morbidity and mortality (10). IEMs frequently manifest with both hyperkinetic (often 2 or more phenotypes) and hypokinetic movement disorders (10). Hyperkinetic movement disorders are unwanted or excess movements which include dystonia, choreoathetosis, tremor, myoclonus, ataxia, tics, and stereotypies. Hypokinetic movements are manifest in akinetic-rigid syndromes or parkinsonism (11). Diseases associated with parkinsonism in adults often present with dystonia in children or young adults (12). Both hyperkinetic and hypokinetic movement disorders are crucial clinical findings with significant implications for diagnosis and treatment. The main IEMs causing movement disorders are neurotransmitter defects, metal-storage diseases (e.g., neurodegeneration with brain iron accumulation [NBIA], Wilson disease), energy metabolism defects, and lysosomal storage disorders (12, 13).

## Study design challenges

### Selection of patient population

There are several challenges related to appropriate patient selection in rare disease clinical trials. A challenge in rare diseases with heterogeneous patient populations is how to select a sufficiently homogeneous patient population for evaluation of treatment response and whether this population is sufficiently representative of all patients with the condition or, in fact, even a majority of patients with the condition. Additionally, selection of a homogeneous population within a heterogeneous rare disease further limits the available population for recruitment and may extend the recruitment period by years. A factor contributing to problems with selecting an appropriate patient population is the rarity of the disease with generally limited available data on incidence and/or prevalence and the limited availability of newborn screening for early diagnosis. The wider availability of whole exome sequencing (WES) next-generation sequencing (NGS) testing will probably greatly facilitate the early and accurate identification of genetic diseases compared with the delays that occur in diagnosis based on observation of the appearance and worsening of symptoms.

The majority of rare diseases, especially IEMs, present along a continuous spectrum of heterogeneity. Generally, once a disease is discovered, the most severe patients are identified first due to obvious greater morbidity and mortality of these phenotypes, which come to the physician's attention first. With time and diagnostic progress, other phenotypes are subsequently discovered. It is common that, initially, phenotypes are strictly defined as severe, attenuated, and intermediate. In reality, there is a full spectrum of heterogeneity, with patients at one end of the spectrum having the most severe disease, at the other end are the most attenuated patients, and in-between, patients present with varying degrees of intermediate phenotypes. The lack of a strong genotype-phenotype correlation results in no clear way to predict disease phenotype and rate of disease progression. Similarly, a lack of biomarkers indicating disease severity or rate of progression makes selection of a homogeneous patient population challenging. Confounding the identification of biomarkers of disease severity is the problem that rarely will these markers be assayable in blood, and cerebrospinal fluid (CSF), which is more difficult to obtain in the context of biomarker studies or natural history, will be required for identification and characterization of these biomarkers. If there were a biomarker proximal to the disease mechanism (i.e., PDAB), upon which accelerated approval of the drug treatment could potentially be based, then a broader patient population with the disease could be enrolled than would be possible with a symptom-based endpoint, and the challenges of selecting the most homogeneous population possible could be avoided (2).

### PKAN example of patient selection challenges and strategies

PKAN is a rare disease and no reliable prevalence data have been collected; accordingly, the true global incidence and prevalence of PKAN are unknown and estimates must be used (14–16). A prevalence estimate of 1–3 in 1,000,000 has been suggested and this figure implies a general carrier frequency of 1:275 to 1:500 (17). As in other rare diseases (18, 19), it should be assumed that some PKAN patients remain undiagnosed or misdiagnosed. The actual number of patients with PKAN may thus be greater than reported. Currently, PKAN is not part of newborn screening panels. The diagnosis of PKAN is usually considered based on clinical suspicion and imaging (i.e., MRI); therefore, the diagnostic delay for PKAN is often substantial, except in patients with a positive family history. Such diagnostic delays may be shortened, including through early screening of asymptomatic siblings of patients, with the increasing availability of relevant WES NGS neurological and neurodegenerative panels for clinical testing. With these panels, patients will continue to be symptomatic when diagnosed; however, the diagnosis may occur earlier, closer to the onset of symptoms, as the treating physician does not need to identify or suspect the diagnosis prior to WES NGS testing.

PKAN is a progressive neurodegenerative disorder clinically affecting movement, balance, speech, vision, cognition, affect, and behavior (20). PKAN covers a continuous clinical spectrum with two major forms generally described in the literature: classic (early onset with rapid progression) and atypical (late onset with slower progression, all non-classic phenotypes), based on the age of onset and disease progression. The hallmark features of classic PKAN include dystonia onset before the age of 10 years, loss of ambulation within 10–15 years of onset, and the main presenting features of overlapping dystonia-parkinsonism syndrome, combined with motor disturbances and early disability, as well as intellectual impairment and retinal degeneration (17, 20). In atypical PKAN, dystonia onset is more common in the second or third decade of life, loss of ambulation may occur within 15–40 years of onset, and the main presenting features include parkinsonism, dysarthria, and dystonia, as well as neuropsychiatric symptoms, which include cognitive decline and behavioral abnormalities (17, 20). Despite this classification, there are patients with early onset but slow progression or late onset with rapid progression, which are often classified as intermediate.

Overall, there is significant heterogeneity both among PKAN phenotypes (classic vs. atypical) as well as within phenotypes, especially among patients with atypical PKAN (17). Further, intrafamilial clinical variability has been described. Classification into classic and atypical PKAN phenotypes is based on clinical features; however, there is no clear way to predict disease phenotype and rate of disease progression at an early age before the onset of disease manifestations such as dystonia, which occurs by the age of 6 years in ~90% of patients with classic PKAN (21). The genotype-phenotype correlation in PKAN is limited. Patients with two null alleles in *PANK2* (which predicts no protein production) show significantly earlier age of disease onset and progress more rapidly to loss of independent ambulation; however, there is significant heterogeneity among patients with other combinations of pathogenic variants, yielding either classic or atypical phenotypes in no predictable pattern (22). Additionally, there are no established disease biomarkers that correlate with disease phenotype in PKAN, and there are no published studies involving potential CSF biomarkers in PKAN (20, 23, 24).

### Strategy: Patient selection

For rare diseases that present with a broad spectrum of heterogeneity, more than one patient cohort or registration study could be considered. This strategy may be particularly needed if there are differences in disease manifestations and rate of disease progression in patient subpopulations, which would require selection of different endpoints and study durations. Although challenging when the number of enrolled patients is low, it may be worthwhile to consider enrichment strategies to decrease heterogeneity and to enhance the ability of the clinical trial to demonstrate a potential treatment effect (25).

In PKAN, classic and atypical phenotypes tend to differ with respect to (1) age of disease onset (i.e., 3–4 years old vs. 13–14 years old, respectively) and, therefore, age of diagnosis as it is generally based on clinical suspicion; (2) initial symptoms and main clinical features (i.e., severe lower limb dystonia with gait abnormalities vs. speech difficulty and neuropsychiatric features, respectively); and (3) rate of disease progression and age of loss of ambulation (i.e., rapid progression with loss of ambulation 10–15 years after disease onset vs. slower progression with loss of ambulation 15–40 years after disease onset, respectively). Because of these differences, two separate studies, one focused on classic patients (with dystonia-relevant endpoints and of shorter duration due to rapid disease progression) and another focused on atypical patients (with speech and neuropsychiatric-relevant endpoints and of longer duration due to slower disease progression) may allow for a better assessment of treatment efficacy. This approach does present challenges related to enrollment of adequately powered patient subgroups in a rare disease that has variable progression rate even within subgroups and has the potential risk that one study may be negative for a treatment effect, with consequent implications for the other study. Rare disease studies with small population enrollment have a rather high risk of randomization of cohorts with differing rates of disease progression into the control vs. treatment group. This so-called “randomization roulette” may dictate the trial result rather than the actual effect of the treatment. This problem obviously becomes more of an issue the more the rare disease group is restricted due to the impossibility of recruiting a sufficiently large cohort for analyses in a reasonable amount of time. Care must be taken to make sure the risk of restricting the enrolled group to increase homogeneity does not create a trial more likely to fail due to inadequate sample size. Alternatively, demonstrating the benefit of an intervention on an endpoint related to symptoms in a heterogeneous patient population increases the risk of a negative study. Balance is needed between enrolling a homogeneous population and a more heterogeneous population. The former is more sensitive to detecting the effect of the intervention; however, the sample size is smaller and the enrollment rate slower compared to the more heterogeneous population.

If a separate cohort approach is used in rare diseases with different phenotypes, such as PKAN, the first study should ideally focus on a population that represents the majority of cases and is generally more homogeneous with faster disease progression, as is the case for the classic subtype of PKAN. It is important to note that generally the earlier the disease begins clinically, the more severe and the faster progression it has, and the therapeutic effect is more difficult to obtain in this patient population. In most rare diseases, including PKAN, the pathological disease process begins in intrauterine life and the earliest possible diagnosis and therapeutic intervention is crucial for the optimal clinical effect.

In the case of a disease phenotype with a later onset and a slower course, a therapeutic effect is more difficult to demonstrate due to the heterogeneity of the disease among patients and the need for longer observation to examine change in clinical endpoints. It is difficult to determine whether the drug slowed down the course of the disease or whether any changes are due to the natural fluctuations of the disease course. With respect to atypical PKAN, patients could be enrolled in a separate study that examines only atypical PKAN or could be examined in disease registries. An alternative would be to conduct an endpoint-enabling study with these patients to establish intrapersonal rates of progression followed by a post-marketing study. The post-marketing study would select the measures identified from the endpoint-enabling study to assess pre- and post-treatment rates of decline in the measures. However, in rare diseases, splitting patients into additional groups may be problematic due to the limited number of patients available for study participation. Some studies in very rare diseases opt to enroll all types of patients, for example an Ultragenyx study in mucopolysaccharidosis (MPS) VII (26), but this may lead to very heterogeneous outcomes and difficulty in data interpretation. In the example of the Ultragenyx study, the enrolled patients had either early or late disease onset but both groups had the same presenting signs and symptoms, and the study excluded patients with severe intellectual disability. The early and late disease patient groups could be pooled due to the only difference being rate of progression. Additionally, enrolling all types of patients may be feasible if the therapeutic approval is based on the correction of a proximal biomarker that truly indexes the core mechanism of the disease (i.e., PDAB). It may also be a reasonable approach if all patients are enrolled in a non-drug follow-up study, which can occur while preparing the Investigational New Drug (IND) submission. Statistical modeling is used to evaluate pre- vs. post-treatment progression rates in each patient and examined across disease severity with different expectations for the extent of the treatment effect for patients with different rates of progression or who are recruited early vs. late in the disease progression.

### Selection of endpoints

In the development of a rare disease clinical trial program and selection of study endpoints, the following should be taken into account: review of the natural history of the disease of interest; review of clinical development programs in the same and other similar rare diseases (both non-investigational natural history and registry studies and investigational studies); engagement with physicians and specialists managing patients with the rare disorder, including 1:1 interviews and scientific advisory board meetings; engagement with patient advocacy groups; engagement with regulatory experts and agencies, including the FDA and EMA, for their input on trial design; interactions with biostatisticians, especially those with experience with regulatory authorities, who can guide novel trial designs; consideration of the conduct of a small exploratory pilot study prior to the pivotal trial; and exploratory studies of biomarkers that may be used as surrogates or outcome measures. Additionally, rare disease trials with a small sample size should collect as much safety data as is possible, and in general there should be particular attention paid to the quality of the data for both efficacy and safety.

Ideally, natural history studies investigate the natural course of a disease from or before inception, through pre-symptomatic, symptomatic, and clinical stages to the point of cure, chronic disease, or death. Understanding the clinical manifestations of the disease over time may identify points of intervention that allow for greater or earlier treatment response in patients and may help to identify relevant study endpoints (25). Natural history studies may provide a clinical baseline and quantify rate and variability of disease progression, though caution should be used with older natural history studies as they may have concentrated on the more aggressive and progressing forms of disease that are identified earlier. Other possible limitations of using natural history studies include poorly defined factors such as environmental changes (including diet) and variability in general aspects of health care over time.

Natural history studies may also aid in detecting safety concerns, provide context for efficacy evaluation or even provide the control cohort (when data are well-collected and matched to the treated population, including genotype-phenotype and ethnicity matching, and the ethics of using a long-duration placebo control group can be questioned), help identify biomarkers or other surrogate measures and determine correlations with disease, guide dose selection, and help establish the optimal window of intervention (27, 28). The results of natural history studies are important for designing clinical trials as well as informing benefit-risk analyses and regulatory decision-making, especially for rare and poorly understood conditions, and historical natural history studies may be accepted by regulatory agencies to use as a control cohort when it would be difficult or unethical to recruit patients for a placebo-controlled study (27, 29). Natural history studies can inform selection of endpoints in rare disease by providing clinical baseline information for areas of intended outcome assessments, by describing the full range of possible disease clinical presentations and possible disease subtypes that may require a different approach to outcome assessments, and by informing the selection or development of sensitive and specific outcome assessments. More information about the role of natural history studies in drug development for rare diseases may be found in the FDA's Draft Guidance for Industry—Rare Diseases: Common Issues in Drug Development (Guidance for Industry) (25).

Engagement with physicians and specialists who treat patients with a rare disease provides informative feedback on the utility and likely tolerability of specific endpoint assessments for patients across the spectrum of clinical presentations. Such discussions with physicians and specialists may help to identify meaningful outcomes for patients related to activities of daily living and quality of life. Additionally, discussions with patient advocacy groups and surveys completed by patients and families are aimed at identifying outcomes that are subjectively clinically meaningful for patient function and quality of life (QOL) (30). Of particular relevance to endpoint selection, survey questions should include the symptoms considered most burdensome and having the most unmet need by patients, their families, and their caregivers. Given the heterogeneity of symptoms in rare diseases, response to treatment may be heterogeneous as well and this should be considered during endpoint selection. The heterogeneity of response may demonstrate the need for composite measures, anchored multi-domain clinical global impression measures, worst problem measures, or goal attainment measures to assess individualized functional changes (31, 32).

Patient Reported Outcomes (PRO) are useful in evaluating clinical trial treatment responses (31, 33, 34); however, they can be time consuming to develop and it may be difficult to demonstrate benefit or improvement using the PRO. Use of a clinical or surrogate primary endpoint that is likely to predict clinical benefit in a pivotal trial is key to identifying treatments with good effectiveness in daily life (35). There have been suggestions to include qualitative patient and caregiver perception of change assessments as well as consideration of goal attainment based on the goals set by individual patients (31, 32). In a rare disease, due to limited available data, qualitative assessments of change by the patient and caregiver provide additional information for the overall assessment of an intervention's benefit and risk. Completion of interviews with patients and families are currently being encouraged by regulatory agencies as an additional method for the assessment of treatment responses and to help determine the thresholds for clinically meaningful change on other measures (31, 32).

### PKAN example of endpoint selection

Endpoint selection in patients with PKAN should consider whether the patients have the classic vs. atypical form. The patient group to be enrolled in the clinical study should be clearly defined and should be indexed by the selected endpoints. The main clinical features in PKAN comprise a variety of neurological symptoms, including movement disorder symptoms such as dystonia, dysarthria, parkinsonism (i.e., rigidity, tremor, bradykinesia, postural instability) and choreoathetosis, pyramidal features such as hyperreflexia and spasticity, intellectual and developmental disabilities, and visual impairment. The main presenting features in classic PKAN include dystonia (e.g., limb, oromandibular [which may result in secondary effects of swallowing difficulty and poor nutrition], and generalized dystonia) with corticospinal tract involvement, intellectual impairment, and retinal degeneration. Patients with atypical PKAN more commonly present with speech-related and neuropsychiatric symptoms followed by later development of milder dystonia or parkinsonism (20). As the disease advances, dystonia and spasticity compromise the patient's ability to ambulate, leading to frequent falls, disability, medical complications, and pain. Most patients with early onset PKAN are wheelchair-bound by their mid-teens (and for many patients, much younger) and patients struggle with multiple losses as their functioning deteriorates through disease progression (24). The brain MRI of patients with PKAN features the “eye of the tiger” sign, which is a central region of hyperintensity surrounded by a rim of hypointensity on coronal or axial T_2_-weighted images of the globus pallidus (36).

The selection of endpoints for a rare disease with neurodegeneration, such as PKAN, includes multiple potential endpoint assessments (Table 2). The types of potential study endpoints within clinical markers and outcome measures may include clinical rating scales, speech and swallowing testing, integrated assessment of balance, gait and physical activity, assessments of neuropsychiatric symptoms and intellectual impairment, and retinal degeneration and neuroimaging endpoints, if a clinical correlation has been described.

**Table 2:** Disease manifestations and corresponding potential endpoints in PKAN and other similar rare diseases presenting with neurodegeneration.

**Disease manifestations**	**Endpoint group**	**Corresponding potential endpoint**	**Details**	**Previous use and comments**
Dystonia and parkinsonism	Clinical rating scales	PKAN-ADL (37)	- Developed specifically for PKAN - Adapted from UPDRS Part II - 12 items of ADL (speech, drooling, swallowing, writing, eating tasks, dressing, personal hygiene tasks, turning or changing position in bed, sitting, falling, walking, and discomfort and pain) - Focused on functional consequences of motor symptoms as the major contributor to impairment	• Primary endpoint in FORT trial (38) • Reached Special Protocol Assessment agreement with FDA on single phase 3 trial evaluating efficacy • Ease of use, brevity • No need for multiple raters • No MCID determined • In FORT trial PBO and active arms saw 1–2 point improvement in 24 weeks
		PKAN-DRS (39)	- Developed specifically for PKAN - Adapted from UPDRS, MDS-UPDRS, UMSARS, BFMDRS, Ashworth Scale - 34 items (maximal score 135) - Encompasses 6 subscales for cognition, behavior, disability, parkinsonism, dystonia, and other neurological signs - Includes both motor and non-motor aspects	• Scale showed good internal consistency and interrater analysis • Discriminated a subgroup of patients with lower scores and c.1583C>T mutation • Requires clinician evaluations of parkinsonism, dystonia, other neurologic signs • Patient-reported subscales completed by patients and caregivers during an interview
		BFMDRS (25, 40, 41)	- Originally established for the clinical assessment of primary torsion dystonia in adults - Two clinician-rated subscales (maximal score 120): a movement subscale in nine body areas (based on patient examination) and a disability subscale of seven items (based on patient's report of disability in activities of daily living)	• Recommended for generalized dystonia • Used in numerous studies to determine treatment effects of DBS (42) • Primary endpoint in pantethine study (43) • MCID has been determined in DBS studies 15–20% in dystonia severity (44)
		UPDRS or MDS-UPDRS Part III (45)	- Developed for the clinical study of Parkinson's disease - Part III is a clinician scored motor evaluation - Most commonly used endpoint in Parkinson's disease trials - Limitations include focus on motor aspects of the condition and lack of an anchor across subscales	• Collected in TIRCON registry • Secondary endpoint in deferiprone (46) and FORT (38) trials • Primary endpoint in pantethine study (43) • MCID has been defined as ± 5 points (47, 48)
	Integrated assessment of static balance, gait and physical activity	Static balance	- Test Conditions: standing upright, double - stance, and semi tandem during eyes-open and eyes-closed condition, each for 30 s - Device: BalanSens^TM^, BioSensics (FDA-registered medical device based on wearables for balance analysis) - Test Duration: 2 min + ~5 min preparation	• Outcomes: area of center of mass sway; ankle sway, ankle/hip strategy during eyes-open and during eyes-closed for each of the test conditions
		Gait	- Test Conditions: assessing walking under three conditions (distance of 10 m): habitual speed, fast speed, and habitual speed while performing a cognitive demanding task (e.g., counting backward from a random number) - Device: LEGSys^TM^, BioSensics (FDA-registered medical device based on wearables for gait analysis) - Test Duration: ~3 min + ~5 min preparation	• Outcomes: gait speed, stride length, stride time, double stance, gait variability, gait initiation, and dual task cost • A relatively objective evaluation may be difficult due to potential extraneous influences, such as patient fatigue or nervousness
		Physical activity	- Test Conditions: remote monitoring of daily physical activity using a validated pendant sensor (PAMSys^TM^, BioSensics) - Device: PAMSys, BioSensics (a validated pendant sensor) - Test Duration: minimum 2 consecutive days; ideally during the entire duration of study (eg, 3 months); sensor preparation: <1 min	• Outcomes: cumulative postures (sitting, standing, walking, and lying); walking characteristics (step count, cadence, longest walking bout); postural transition (number and duration of sit to stand and stand to sit); activity behavior (sedentary activity, light activity, moderate to vigorous activity), sleep quality (time in bed, sleep duration, sleep efficiency)
		Falls	- Test Conditions: Remote monitoring of falls and daily steps using a validated pendant sensor (PAMSys Fall^TM^, BioSensics) - Device: PAMSys Fall, BioSensics (a validated pendant sensor) - Test Duration: Minimum 4 weeks; ideally during the entire duration of study (e.g., 3 months); sensor preparation: <1 min	• Outcomes: fall time, steps per hour, number of falls per week or month, number of falls per steps
Dystonia (oromandibular)	Speech and swallowing testing	Speech breathing	- Domain: Respiratory function - Test Conditions: participants are instructed to read a short paragraph at their normal rate and volume. The reading is recorded using a camcorder and analyzed in the lab. - Device: video recorder and headset microphone - Test Duration: 5 min	• Outcomes: an automated Matlab script, SPA is used to derive measures of speaking rate, and percentage of pausing
		MPT and phonatory stability	- Domain: Phonatory function - Test Conditions: Participants are asked to take a deep breath and to say “ah” for as long as they are able on one breath. The sustained “ah” is recorded using a camcorder and later analyzed in the lab - Device: video recorder and headset microphone - Test Duration: 3 min	• Outcomes: automated algorithms in Praat are used to extract voice perturbation measures (e.g., jitter, shimmer, HNR, etc.) and duration measured in seconds
		DDK rate	- Domain: articulatory function - Test Conditions: participants are asked to take a deep breath and to repeat the syllables “ba” (to measure labial movement), and “badaka”, as quickly and accurately as they can on a single breath. The productions are recorded and later analyzed in the lab. - Device: video recorder and headset microphone - Test Duration: 10 min	• Outcomes: an assessor in the lab counts the total number of syllables produced per second manually • Many patients, especially younger patients, are not able to perform the DDK assessment
Neuropsychiatric symptoms and intellectual impairment	Cognitive, behavioral, and functional outcomes testing	Neurocognition	- Bayley Scales of Infant and Toddler Development - Mullen Scales of Early Learning - Age-based Weschler Intelligence Scales (WPPSI, WISC, WAIS) - NIH toolbox Cognition Battery - Cambridge Neuropsychological Test Automated Battery (CANTAB) - Stanford Binet Intelligence Scales	• For all measures, important to be aware of the motor component and whether the test is valid with a motor disability • Bayley Scales may not be relevant due to patient age at diagnosis • WPPSI may be too difficult to complete for patients with severely impaired speech and intellectual impairment • NIH Toolbox has adaptations and validation for intellectual disability, and is a repeatable battery administered on an iPad, which may make it easier to perform for those with motor disability • CANTAB requires good eyesight vs. patients may have retinal degeneration and dystonic eye movements and is challenging for individuals with intellectual impairment • Stanford Binet has change-sensitive scores and Z-deviation method to address floor effects in scoring
		Adaptive behavior	- Vineland Adaptive Behavior Scale (VABS)	• Is accepted as a well-validated adaptive behavioral measure • The FDA is familiar with VABS
			- Adaptive Behavior Assessment System II (ABAS-II)	• Considered a good measure of functional outcomes • Working toward FDA recognition of ABAS-II
Retinal degeneration^a^	Neuro-ophthalmologic examination and electroretinogram (ERG)	Neuro-ophthalmologic examination	- Snellen acuity - Hardy-Rand-Rittler color plate testing - Ocular motility - Convergence - Visual fields (Goldmann perimetry) - Photographs of the fundus - Optical coherence tomography - Ret-EVAL: retinal function/ERG marker	• Snellen acuity requires cooperation and speech • Ocular motility, convergence, and visual fields are difficult to quantify • Ret-EVAL can be completed in the clinic in a few minutes, although does require cooperation from the examinee
Brain iron accumulation^b^	Neuroimaging	MRI	- Pros: non-invasive - Cons: reductions in iron are unlikely to be impacted by mechanism of action of most drugs; monitoring longitudinal changes in iron will take a long time	• PKAN patients exhibit characteristic eye of the tiger iron accumulation in the globus pallidus • Treatment with deferiprone decreased mean iron concentration in globus pallidus as measured by MRI-R2^*^ (46)
		CT	- Pros: non-invasive - Cons: not a universal phenotype, and may be influenced by sex; latency to treatment effect unknown, but may be long; radiation exposure during CT	• Calcium accumulation in the globus pallidus was observed in 47% of PKAN patients (*n =* 15) (49) • 75% of patients with phenotype were female
		MRS	- Pros: non-invasive - Cons: unknown utility; technical concerns; no methods developed for clinical use; high variability; lack of reproducibility	• Reduced Glx and NAA observed by MRS in neuronal *PanK1,2* KO mice was fully and partially recovered with BBP-671 (50) • Reduction of both Glx and GABA by MRS suggests impaired excitatory and inhibitory neuronal metabolism in *PanK1,2* KO mice that can be rescued by BBP-671 (50)

BFMDRS, Burke-Fahn-Marsden Dystonia Rating Scale; CT, computed tomography; DBS, deep brain stimulation; DDK, diadochokinetic; GABA, gamma-aminobutyric acid; Glx, glutamate+glutamine; HNR, harmonics-to-noise ratio; KO, knock-out; MCID, minimal clinically important difference; MPT, maximum phonation time; MRI, magnetic resonance imaging; MRS, magnetic resonance spectroscopy; NAA, N-acetyl aspartate; NIH, National Institutes of Health; PBO, placebo; PKAN-ADL, Pantothenate Kinase-Associated Neurodegeneration Activities of Daily Living; PKAN-DRS, Pantothenate Kinase-Associated Neurodegeneration Disease Rating Scale; Praat, computer software package for speech analysis; SPA, speech pause analysis; UMSARS, Unified Multiple System Atrophy Rating Scale; UPDRS, Unified Parkinson's Disease Rating Scale; WAIS, Wechsler Adult Intelligence Scale; WISC, Weschler Intelligence Scale for Children; WPPSI, Wechsler Preschool and Primary Scale of Intelligence.

^a^Other relevant organ endpoints in rare diseases may include, for example, liver, kidney, and heart function.

^b^Other relevant neuroimaging assessments in rare diseases may include, for example, volumetric assessments and white matter assessments.

Several clinical rating scales for the assessment of dystonia, a prominent clinical manifestation in PKAN, are available and the selection should be based on whether the measure evaluates outcomes considered to be clinically meaningful to patient function and QOL. PRO clinical rating scales developed specifically for the rare disease, if available, may be selected as primary or secondary endpoints and this approach is consistent with current regulatory guidance on clinical trial outcomes. In PKAN, two such scales are available, the PKAN-ADL (37) and the PKAN-DRS (39) (Table 2).

For rare diseases that affect speech and/or swallowing, speech-related outcomes can be assessed (e.g., speech assessments and objective dysarthria rating in patients with progressive supranuclear palsy [NCT04753320] and speech assessment in patients with ALS [NCT05271435]). When selecting an assessment approach, the need for cognitive skills, reading fluency, absence of delayed speech, or requirements for a specialist to implement the assessment should be considered. In practice, a relatively objective evaluation of speech may be difficult due to the potential extraneous influences of patient fatigue or nervousness. Showing a clinically meaningful change in speech function, that patients and families can appreciate, may not be a reasonable expectation; however, quantifying speech may show an improvement, or a lack of further decline relative to pre-treatment assessments, that supports treatment is having a positive or stabilizing effect.

Wearable technology for movement assessment allows for objective “naturalistic” collection of data when patients are at home. This overcomes the more artificial environment of the clinic that is further impacted by greater fatigue due to long-distance travel to clinic appointments. Potential wearable technology outcomes can be evaluated for inclusion as exploratory outcomes, to contribute to the field of knowledge in the rare disease and to inform future trial development. Integration of wearable technology into clinical studies to assess movement (e.g., gait, balance, and physical activity in patients with progressive supranuclear palsy [NCT04753320], chorea in patients with Huntington's disease [NCT03599076], and bradykinesia, tremor, rigidity and postural instability in Parkinson's disease [NCT03100149]) as well as to identify potential digital biomarkers of disease progression (e.g., motor functions and falls in amyotrophic lateral sclerosis; NCT05271435) using remotely-supervised assessments as well as fully remote assessments are underway. Similar to speech assessments, a relatively objective evaluation in the clinic may be difficult due to potential extraneous influences, such as patient fatigue or nervousness.

Retinal degeneration can be assessed as an endpoint by taking photographs of the fundus or using optical coherence tomography at individual stages of the clinical trial to compare the changes over the course of treatment. Examination of neuroimaging markers to indicate disease severity, disease progression, or response to treatment requires longitudinal study and comparison of patients with the rare disease with matched healthy control patients. The potential usefulness of MRI, CT, and MRS in clinical trials involving pediatric patients is currently limited beyond their use for safety assessments, as in hydrocephalus with intrathecal antisense oligonucleotide treatment.

### Strategy: Endpoint selection

Endpoint selection may be relatively straightforward through selection of the most significant central nervous system (CNS) sign or symptoms for the disease, or a milder sign or symptom in a patient study group from which the most significant sign or symptom has been excluded. However, for many rare diseases, well-characterized efficacy endpoints and their assessment, appropriate for the specific disease, are not available. IEM movement disorders include several signs and symptoms. Some of these include (1) diffuse clinical picture with several neurological and non-neurological signs; (2) combination of different movement disorders; (3) distinct neuroradiological findings; and (4) family history (10). Many IEMs present with a mixed movement disorder, often in combination with accompanying neurological, psychiatric, or visceral manifestations (51). With disease progression, more movement disorders may develop, leading to complex and mixed phenotypes, and the dominant movement disorder may be replaced by another movement disorder that may become dominant over time (51). Dystonia is a major symptom in many different metabolic diseases, and almost all neurometabolic disorders can cause dystonia at some stage (12). Although other signs of neurological dysfunction usually coexist, some metabolic diseases can initially present with isolated dystonia (e.g., PKAN, Leigh syndrome, juvenile form of metachromatic leukodystrophy, selected gangliosidosis) (12). In young children (older than 2 years), NBIA cause progressive dystonia and bulbar symptoms and pyramidal signs whereas parkinsonism usually appears in later years (13, 52). Cognitive decline occurs in some NBIA subtypes, but often cognition is relatively spared. Parkinsonism as the leading sign can begin in childhood in, for example, lysosomal disorders, ceroid lipofuscinosis, Niemann Pick Disease Type C (NP-C), and Wilson disease; however, it is more common to begin during adolescence or adulthood (13, 52). For diseases that present with a broad spectrum of heterogeneity and with differences in disease manifestations and rate of disease progression in patient subpopulations, such as PKAN, patients may need to be separated into more than one cohort or study with different endpoint selections.

### Study duration

Though challenging with slowly progressing and highly variable rare diseases, the selected study duration ideally should allow for assessment of clinically meaningful outcomes and capture disease course variability, which may be discovered using natural history data when available (25). When designing a clinical trial, determinants of study duration are the selected primary and secondary endpoints. Study duration should account for when the clinical presentations of interest (i.e., milestones or changes in rating scales that are related to the study endpoints) are likely to develop and/or persist. Determination of the study duration also considers whether the treatment being evaluated is expected to exclusively slow progression of the disease or if the treatment is expected to improve symptoms, and the estimated time course for each of these outcomes. Among rare neurodegenerative diseases, if the treatment is being started very early in the disease onset, the potential for symptom improvement (in addition to slowing progression) is greater as dysfunctional neurons that can be rescued with treatment may still be present. If the treatment is initiated later in the disease course, following greater loss of neurons, slowing of progression is more likely to be the achievable outcome. Estimating the amount of treatment effect that may provide clinically meaningful benefit, whether symptom improvement or stabilization, and the time to achieve the minimally important difference in the study patient population, will inform the study duration. In rare neurodegenerative diseases with slow progression and high variability, study duration to show clinically meaningful change may not be feasible due to the need for a several-years randomized controlled trial. Alternatively, with identification of a PDAB, a shorter controlled trial may support accelerated approval and be followed by a longer post-marketing study to show clinical benefit (2).

### PKAN example: Study duration decision

The pattern of progression of classic PKAN (up to 80% of patients with PKAN) is saltatory, with stepwise decline occurring over a few days or weeks, followed by plateauing that may be sustained for weeks or months (20). The overall trajectory of change is that of relentless progression, and no clear exacerbating factors have been identified to account for the periods of more precipitous decline (20). This pace continues until the child loses all independent function, typically during the second decade of life. Atypical PKAN generally progresses more slowly, and some individuals experience plateauing of symptoms for many months, years, or even decades. Static disease or minimal progression over 5–10 years is not uncommon for patients with atypical PKAN, making this patient population particularly challenging to study in a clinical trial setting. Longer-term treatment in patients with PKAN may be necessary to observe a significant and meaningful treatment benefit due to the natural pattern of progression with the disease. For example, in the FORT study that examined a potential new treatment (i.e., fosmetpantotenate) in patients with PKAN (Table 3), only a modest decrease in health and functioning (1–2 points) from baseline to 24 weeks using the PKAN-ADL was seen in the placebo group and the change for the mean PKAN-ADL score in the fosmetpantotenate group was not statistically significantly different from the change in the placebo group (38). The FORT trial duration may not have been long enough for a robust evaluation of the treatment given the heterogeneity of disease among the enrolled patients and the level of sensitivity to change in the PKAN-ADL.

**Table 3:** Lessons learned from pivotal trials in PKAN and other neurodegenerative genetic disorders.

**Drug/company**	**Design, inclusion criteria, duration**	**Endpoints**	**Outcome**	**Comments on study design**
PKAN Deferiprone (iron chelator) (46); ApoPharma USA, Inc.	• Randomized, double-blind, placebo-controlled • Duration: 18 months • *N* = 88 patients (58 deferiprone, 30 placebo) • Inclusion criteria: ✓ 4 years and older ✓ Genetically confirmed PKAN ✓ A score ≥3 on the BAD Rating Scale • Stratification based on age group at onset of motor symptoms • Followed by 18-month, single-arm, open-label extension	• Co-primary endpoints: ✓ BAD scale ✓ PGI-I scale • Secondary endpoints: ✓ Parts I, II, III and IV of UPDRS ✓ FIM/WeeFIM ✓ PedsQL ✓ PSQI ✓ Iron concentrations in the globus pallidus (MRI-R2^*^)	• Primary endpoints: not met, indication of a decrease in disease progression • Secondary endpoints: effective in lowering iron levels in the brain as measured by MRI-R2^*^	• Broad patient population • Uncertain whether lowering iron levels is associated with disease modification or removing iron ions which are not the cause of disease in already dead tissue
PKAN Fosmetpantotenate (phosphopantothenate replacement therapy, FORT) (38, 53); Retrophin, Inc.	• Phase 3 randomized, double-blind, placebo-controlled, multicenter • Duration: 24 weeks • *N* = 84 patients (41 fosmetpantotenate, 43 placebo) • Inclusion criteria: ✓ Age 6–65 years ✓ Confirmed pathogenic variants in the PANK2 gene ✓ A score of ≥6 on the PKAN-ADL scale • A score of ≥6 on the PKAN-ADL indicates at least mild impairment across multiple functional domains or moderate-to-severe impairment in at least 1 domain	• Primary endpoint: PKAN-ADL • Secondary endpoint: UPDRS III total score • Exploratory endpoints: ✓ BAD scale ✓ CGI-I scale ✓ Quality of Life in Neurological Disorders adult and pediatric versions ✓ EQ5DY ✓ FIM/WeeFIM ✓ 25-foot walk test ✓ DDK speech assessments	• Primary endpoint: not met • Secondary endpoints: not met	• Broad patient population • Short study duration • PKAN-ADL may not be sensitive enough to measure small improvement • BAD scale has limitations in dystonia assessment • EQ5DY has limitations for QOL assessment • FIM/WeeFIM is complicated to use • Few pediatric patients can perform 25-foot walk test and DDK speech assessments
NP-C Miglustat (substrate reduction therapy) (54, 55); NCT00517153 Actelion	• Phase 2 randomized, double-blind, placebo-controlled • Duration: 12 months • *N* = 29 • Inclusion criteria: ✓ Age 12 years or more (pediatric sub-study 4-11 years)	• Primary endpoint: HSEM velocity (HSEM-α and HSEM-β) • Secondary endpoints: ✓ Swallowing ✓ Auditory acuity ✓ Ambulatory ability (standard ambulation index) ✓ Cognition (MMSE)	• Primary endpoint: improved HSEM velocity • Secondary endpoints: improved swallowing, stable auditory acuity, slower deterioration ambulation in patients older than 12 years	• HSEM velocity selected as primary endpoint based on correlation with disease progression
NP-C Arimoclomol (56)**;** NCT02612129 Orphazyme	• Phase 2/3 randomized, double-blind, placebo-controlled, multicenter • Duration: 12 months • *N* = 50 • Inclusion criteria: ✓ Age 6–18 years (pediatric sub-study 24 months−6 years) ✓ Confirmed pathogenic variants in the *NPC1* or *NPC2*	• Primary endpoint: NP-C clinical severity score (NPCCSS) • Secondary endpoints: ✓ NPC-cdb score ✓ NPCCSS score ✓ NPCCSS score (individual domains) ✓ EQ5DY ✓ SARA score ✓ 9-HPT ✓ CGI-S ✓ CGI-I	• Primary endpoint: significant reduction in annual disease progression • Secondary endpoints: proportion of responders analysis of NPCCSS, NPC-cdb, CGI-I not significantly different	• Treatment effect was increased in subgroups of patients well-balanced in disease characteristics
CLN2 rhTPP1, cerliponase alfa (ICV ERT) (57); BioMarin Pharmaceutical	• Phase 1/2 open-label dose-escalation • Duration: 12 months • *N* = 24 • Inclusion criteria: ✓ Age 3– <16 years old ✓ Diagnosis of CLN2 by TPP1 enzyme activity (dried blood spot) ✓ Mild to moderate disease: 2-domain score of 3–6 on Hamburg Scale motor and language domains (score of at least 1 in each domain)	• Primary endpoints: ✓ AEs ✓ Motor-Language scale • Secondary endpoints: ✓ MRI: whole brain volume ✓ MRI: volume total gray matter ✓ MRI: volume total white matter ✓ MRI: volume of CSF ✓ MRI: whole brain apparent diffusion coefficient ✓ Brain atrophy ✓ Immunogenicity of study drug in CSF and serum	• Primary endpoints: less decline in motor and language function than historical controls; serious AEs of failure of intraventricular device and device-related infections • Secondary endpoints: not reported	• Historical control comparison
CLN2 rhTPP1, cerliponase alfa (ICV ERT) (58) NCT02678689 BioMarin Pharmaceutical	• Phase 2 open-label, multicenter • Duration: 144 weeks • *N* = 14 • Inclusion criteria: ✓ Age <18 years ✓ Diagnosis of CLN2 disease by TPP1 enzyme activity (dried blood spot) in the fibroblasts and leukocytes ✓ Score 3–6 Hamburg CLN2 motor-language scale	• Primary endpoints: ✓ AEs ✓ Motor/Language score on the Hamburg CLN2 rating scale ✓ Immunogenicity of study drug in CSF and serum • Secondary endpoints: ✓ Total Hamburg CLN2 rating scale ✓ Clinical laboratory tests ✓ Neurological examinations ✓ MRI change in brain volumes ✓ Time to disease manifestation in asymptomatic patients	• Not yet reported	

AEs, adverse events; BAD, Barry-Albright Dystonia; CBD, clinical database; CGI-I, Clinician Global Impression of Improvement; CGI-S, Clinician Global Impression of Severity of Illness; CLN2, neuronal ceroid lipofuscinosis 2; CSF, cerebrospinal fluid; DDK, Diadochokinetic; EQ5DY, EuroQoL 5-dimension 3 level and youth versions; ERT, enzyme replacement therapy; FIM, Functional Independence Measure; 9-HPT, 9-hole peg test; HSEM, horizontal saccadic eye movement; ICV, intracerebroventricular; MMSE, mini-mental status examination; MRI, magnetic resonance imaging; NP-C, Niemann Pick Disease Type C; PKAN-ADL, Pantothenate Kinase-Associated Neurodegeneration Activities of Daily Living; PGI-I, Patient Global Impression of Improvement; PedsQL, Pediatric Quality of Life; PSQI, Pittsburgh Sleep Quality Index; rhTPP1, recombinant human tripeptidyl-peptidase 1; SARA, Scale for Assessment and Rating of Ataxia; UPDRS, Unified Parkinson's Disease Rating Scale; WeeFIM, Functional Independence Measure for Children.

### Strategy: Study duration

An important consideration when deciding study duration is understanding what is possible within the rare disease patient sample. If the study will be placebo-controlled, a duration longer than a year in a neurodegenerative disease will likely not be acceptable to families and can be argued as unethical, particularly when unable to predict which patients will lose function quickly versus more slowly. However, other than studies that use a surrogate biomarker or biomarker showing reversal of the core mechanism of disease, study duration longer than a year will likely be needed for robust assessments of treatment outcomes. To accomplish this ethically, study designs should include natural history control groups, case-controls from natural history, or within patient pre- and post-treatment comparisons of trajectory of decline with a measure(s) that can detect rate of decline across the disease course. Within the context of good safety outcomes associated with the treatment and very slowly progressing disease, long-duration follow-up with assessment of milestones as important endpoints may allow patients to be followed for longer periods.

Another possibility when determining the study duration is to use varied study duration. In this approach, there is continued follow-up of patients up to a maximum period (e.g., 1 year) for patients enrolled earlier in the study while the last enrolled patient completes the minimum amount of follow-up (e.g., 6 months). While the efficacy outcomes for all patients would be evaluated at 6 months, follow-up data between 6 and 12 months for those who enrolled earlier would provide additional efficacy data. This concept of varied study duration is further discussed below in the Biostatistical considerations Section.

### Choice of control groups

Ideally, the goal is to obtain an unbiased estimate of the effect of the treatment being investigated compared to placebo, another active compound, or standard of care (5). A comparative trial will usually be preferrable but may not always be possible. In general, there are two approaches to selecting control patients, internal controls (i.e., among the patients enrolled in the study, these patients are randomly assigned to placebo or standard of care treatment) or external controls (i.e., these patients were evaluated outside of the patients enrolled in the study, such as in a natural history group), and these patients may be historical or concurrent (5). Although internal controls are the preferred option for comparative trials, under exceptional circumstances external controls may be acceptable (5). In rare disease trials with small sample sizes that use clinical endpoints, the patient may serve as their own control, with a comparison of the pre-treatment versus post-treatment trajectory. Additionally, a pre-treatment run in period can be beneficial as it may reduce the placebo effect and provide a more stable baseline measurement, which increases the power for analyses of change from baseline. Use of a crossover design is likely not feasible due to the long treatment duration and lack of understanding of potential carryover effects which could interfere with the identification of a true treatment effect by diminishing any differences between crossover study drug conditions.

The use of historical control designs is generally restricted to assessment of serious disease when: (1) there is an unmet medical need; (2) there is a well-documented, highly predictable disease course that can be objectively measured and verified, such as high and temporally predictable mortality; and (3) there is an expected drug effect that is large, self-evident, and temporally closely associated with the intervention (25).

### Biostatistical considerations

In rare diseases with small and very small populations, less conventional or less common study designs and analytic approaches may be used to improve the interpretation of study results (5). Well-planned and well-controlled, blinded, randomized trials continue to provide the highest evidence in drug development clinical trials. A trial with 1:1 randomized allocation is most efficient to assess efficacy. However, an unequal randomized allocation, such as 2:1 (with a greater number of patients exposed to investigational treatment), is often a consideration to increase the size of the safety database. Stratification by a few important prognostic variables is important to minimize the risk of imbalance, and, although difficult to accomplish beyond 1 or 2 variables in most rare disease populations, is of greater benefit in trials of small size. Adaptive study designs can play a critical role by allowing the trial to adjust according to unblinded interim data obtained from the trial itself. This may be challenging to implement in a rare disease that requires a long duration (e.g., >6 months) before a treatment response can be demonstrated. With adaptation, often corrective measures are necessary to ensure that the Type I error probability is not inflated (59). Although trials are typically powered at a two-sided Type I error rate equal to 5%, for rare diseases with a very limited patient population, a less stringent threshold may be acceptable (60). This allows a trial to be adequately powered with a smaller sample size.

Critical questions related to statistical analyses in rare disease clinical trials include sample size, disease characteristics to measure, and study duration to be able to achieve robust evaluation of change in response to treatment in the primary endpoint and other important endpoints. To address these challenges, innovative study designs and statistical analyses continue to be developed and examined (61).

Of special interest are study designs and analysis methods to increase power in small population studies. In the case of very rare and heterogeneous disease, enrolling even a small patient sample (e.g., <50 patients) may take up to two or more years, and the small population may limit stratified allocation to one variable. Within these conditions, the disease characteristics of the control group and treated group may be imbalanced at baseline such that one or the other patient group is expected to progress faster. This is potentially further exacerbated when using 2:1 randomization (which also reduces power), can adversely affect power, and may result in a failed trial. The analysis method can adjust for some of the imbalance, but the risk of a failed trial remains, even if the drug treatment appears to show efficacy based on natural history, within-patient analyses, and/or exploratory biomarker endpoints. Due to the rarity of the disease, there is a real possibility that the trial cannot be re-run. Thus, every effort should be made to optimally design trials or develop analysis methods that give the investigational drug the best chance of robust evaluation. If a disease is sufficiently rare and heterogeneous as to be unable to statistically create a feasible well-controlled, blinded trial, then a different type of study design is needed.

### Strategy: Biostatistical considerations

One proposal to improve the chance of success for rare disease trials is combining results across multiple methods of analysis. For pivotal trials, the convention is to prespecify a single method of analyzing the primary endpoint. Reliance on a single method is risky as the model selected for analysis may be suboptimal. In rare disease trials with a small sample size and limited experience with endpoints, this risk is of particular concern. A more robust approach is to combine alpha-adjusted *p*-values from prespecified multiple methods of analysis. One such approach is to choose the smallest *p*-value from, say, 3 pre-specified methods of analysis. The adjusted alpha for selecting the best *p*-value is obtained via the permutation procedure. Compared to the conventional method, this approach provides more power than a single suboptimal method, with minimal loss in power if the single method of analysis is optimal (62–64).

## Clinical trial recruitment

Emerging treatment trials are competing for small populations of patients with rare diseases. Among the patients with the most severe phenotype and high mortality (e.g., in PKAN, the classic phenotype) patients may be critically ill and not meet inclusion criteria for clinical trial participation. Additionally, these patients may be more likely to drop out of the trial due to the level of their disability and difficulty meeting the rigor of the study requirements over time. To help meet the challenges of traveling to study visits for these patients, home visits may be needed. If an ongoing trial has enrolled most of the available patients with the rare disease within a geographic region, a new trial will need to shift to a different geographic location for study enrollment. This may introduce challenges in the consistent conduct of the clinical trial, especially in countries that may be less experienced in the conduct of randomized clinical trials. Otherwise, for typical inclusion and exclusion clinical trial criteria (i.e., no prior enrollment in a clinical trial of an investigational product within 30 days prior to randomization), patients participating in a trial would have to stop participation and stop treatment for a period to be eligible for a new study, and it is unlikely patients would choose to do so. In some rare diseases, the treatments being examined may center on a similar biochemical pathway and failure to benefit from a drug that targets the pathway may signal likely poor outcomes with other drugs targeting the same pathway.

Patients and families with rare disease show high interest in clinical trial participation, as seen in the large patient enrollments in the deferiprone and fosmetpantotenate clinical trials for treatment of PKAN (38, 46). Patient enrollment hesitancy may occur based on the duration of the clinical trial, access to the trial treatment, and the amount of travel required for study visits, and this must be considered when designing short-term and long-term studies and the addition of an open-label extension in which all patients receive treatment. Notably, inclusion of an open-label extension further reduces available patient candidates for subsequent clinical trials. Additionally, typically, patients must be willing and able to remain on their pre-study dose of any allowed concomitant medications (e.g., symptomatic therapies) for the duration of the double-blind period of the study. It is possible that during the clinical trial a patient may develop a new symptom *de novo* or experience worsening of a pre-existing symptom that requires other therapeutic intervention, that then may compromise the assessment of the clinical trial therapy. For example, it is possible that a patient with PKAN could show progression in dystonia to the point that deep brain stimulation might be considered and the symptomatic benefit from this intervention could interfere with the evaluation of the clinical outcome measures, particularly in the absence of disease severity biomarkers.

## Importance of regulatory feedback

Regulatory agencies strongly advise early and continuous communication related to scientific advice and protocol assistance in clinical trials involving small populations (5, 65). Input from multiple regulatory agencies is highly recommended during the design of small population clinical trials (66). Regulatory guidance precedence with rare disease clinical trials allows for greater flexibility in study design and analyses; however, potential drawbacks in using such approaches should be considered early in the clinical development program (67). Inclusion of controls and comparator groups in rare disease clinical trials remains very important for interpretation of study outcomes (5, 25), both from the perspective of evaluation of efficacy and evaluation of safety. Study designs that use the patient as their own control need more evaluation and consideration by regulatory agencies as acceptance of their implementation could potentially help meet several challenges in trials with rare disease populations.

As articulated by Billy Dunn, MD, Director of the FDA Office of Neuroscience, during a September 7, 2022 advisory committee meeting reviewing a drug for amyotrophic lateral sclerosis, there is a duty to use the broadest possible regulatory flexibility while reviewing new drug applications to treat serious, severely debilitating, and life-threatening diseases with substantial unmet need (68). Dr. Dunn stated that the use of broadest regulatory flexibility in these situations acknowledges “the need to tolerate a greater degree of residual uncertainty when making a subjective decision on approval”. This tolerance prioritizes the acceptability of potential risk of a false positive conclusion of approving a drug that later is shown to not be effective over the risk of a false negative conclusion of rejecting a drug that may be effective. Uniform application of this broadest regulatory flexibility is needed for severely debilitating and life-threatening diseases with substantial unmet need, including in the consideration of potential new drugs for rare neurodegenerative diseases, to allow for mechanism-targeted treatments and early intervention for patients.

There is a strong need for multinational collaboration in the study of rare diseases, and multinational clinical trials in rare diseases have unique challenges, including limitations of endpoints delivered in multiple languages and the presence of non-harmonized regulatory requirements across countries (69). The current state of the regulatory environment for multinational rare disease clinical trials may pose challenges in the ability of investigators to collaborate and initiate participation of the clinical trial centers.

Discussions with regulatory liaisons for their feedback on multicenter and multinational clinical trial design planning in rare disease play an important role in study design decision-making. Feedback should be sought early and throughout the clinical trial program development. The regulatory agency emphasis on inclusion of study endpoints that assess a clinically meaningful impact of treatment on patient function and QOL in the real-world informs the selection of the primary and secondary endpoints and the assessment of the effectiveness of the drug treatment of CNS neurodegenerative rare diseases.

## Lessons learned from other trials

The study design decisions and outcomes in prior clinical trials in the rare disease of interest can be examined for relevant information on likely challenges and areas that may need alternative strategies during development of a clinical trial program for a new treatment (Table 3). Two such trials in PKAN are the deferiprone and fosmetpantotenate treatment trials (38, 46). Examples of trials in other rare neurodegenerative genetic diseases include NP-C (54, 56, 70) and neuronal ceroid lipofuscinosis 2 (CLN2) (57).

## Relevance and application to other rare pediatric diseases with neurodegeneration

Rare diseases with features similar to PKAN (i.e., no identified biomarker, often delayed diagnosis, heterogeneous patient population) have similar clinical trial development challenges (Table 4 provides selected examples). Rare diseases with contrasting features, such as an identified biomarker and early diagnosis *via* newborn screening, do not face the same challenges as occur in PKAN. These types of rare diseases have a more straightforward approach to diagnosis, treatment, and assessment of treatment outcomes, illustrating that the challenges within the rare disease space are not uniform. Disease-modifying therapies already exist for a number of IEMs, and early recognition and treatment can prevent irreversible CNS damage and reduce morbidity and mortality (71). Understanding of some pediatric movement disorders is rapidly evolving, stimulated by progress in the discovery of monogenic neurogenetic disorders and, consecutively, the development of structured diagnostic and therapeutic approaches. It is possible that this gap in challenges and the currently evolving progress in some areas may eventually be narrowed by the availability of WES NGS and the possibility of earlier diagnosis across these rare diseases.

**Table 4:** Selected rare pediatric diseases and inborn errors of metabolism presenting with movement disorders.

**Disease group**	**Disease examples (gene, enzyme/protein, OMIM)**	**Clinical features**	**Imaging and laboratory investigations (diagnostic metabolites)**	**Treatment**
Iron toxicosis	Aceruloplasminemia (*CP*, ceruloplasmin)	• Cerebellar ataxia • Dystonia, dysarthria, tremors, chorea, rigidity • Retinal degeneration • Endocrine abnormalities (diabetes mellitus) • Anemia	• MRI: iron accumulation in GP, putamen, caudate, thalamus, RN, dentate, cerebellar atrophy • Labs: microcytic anemia, serum Fe↓, serum ferritin↑	• Iron-chelating therapy • Zinc sulfate
	Neuroferritinopathy (*FTL*, ferritin light chain protein)	• Chorea, dystonia, tremor, bradykinesia • Cerebellar ataxia • Cognitive decline	• MRI: iron accumulation in GP, SN, caudate, putamen, cystic changes and cavitation in caudate and putamen • Labs: serum ferritin↓	• No therapeutics approved • Supportive measures and symptom-based interventions
Rare classical forms of NBIA	PKAN (*PANK2*, pantothenate kinase 2, #234200)	• Dystonia-parkinsonism • Spasticity, hyperreflexia, extensor toe signs • Retinal degeneration • Neuropsychiatric symptoms	• MRI: iron accumulation in GP and SN, eye-of-the-tiger sign	• No therapeutics approved • Supportive measures and symptom-based interventions
	MPAN (*C19orf12*, mitochondrial membrane protein)	• Spasticity (more prominent than dystonia) • Motor neuropathy • Cognitive decline • Optic atrophy	• MRI: iron accumulation in GP and SN, cerebellar and cortical atrophy, T_2_-weighted images can be mistaken for eye-of-the-tiger sign	• No therapeutics approved • Supportive measures and symptom-based interventions
	Kufor-Rakeb syndrome (*ATP13A2*)	• Parkinsonism • Dementia • Neuropsychiatric changes	• MRI: variable GP or no iron accumulation, cerebral, cerebellar, and brain stem atrophy	• No therapeutics approved • Supportive measures and symptom-based interventions
	CoPAN (*COASY*)	• Dystonia-parkinsonism, spasticity • Cognitive decline • Obsessive-compulsive behavior	• MRI: iron accumulation in GP and SN	• No therapeutics approved • Supportive measures and symptom-based interventions
	Woodhouse-Sakati syndrome (*DCAF17*)	• Dystonia, dysarthria • Cognitive decline • Endocrine abnormalities (hypogonadism, alopecia, diabetes mellitus)	• MRI: iron accumulation in GP, white matter disease	• No therapeutics approved • Supportive measures and symptom-based interventions
	FAHN (*FA2H*, fatty acid hydroxylase, #612319)	• Ataxia, dystonia, dysarthria, tetraparesis • Optic atrophy • Seizures • Cognitive decline	• MRI: iron accumulation in GP and SN, pontocerebellar atrophy, diffuse cerebral atrophy, thinning corpus callosum, optic atrophy	• No therapeutics approved • Supportive measures and symptom-based interventions
	PLAN (*PLA2G6*, #603604)	• Dystonia-parkinsonism, ataxia, tetraparesis • Cognitive decline • Optic atrophy • Eye movement abnormalities	• MRI: variable GP and SN iron distribution or no iron accumulation, cerebellar atrophy and optic atrophy are hallmark features	• No therapeutics approved • Supportive measures and symptom-based interventions
	BPAN (*WDR45*, beta-propeller protein, #300894)	• Seizures • Abnormal behavior similar to ASD and Rett syndrome • Dystonia-parkinsonism • Dementia	• MRI: early childhood hypomyelination and thin corpus callosum, iron accumulation in SN>GP	• No therapeutics approved • Supportive measures and symptom-based interventions
Copper toxicosis	Wilson disease (*ATP7B*, transmembrane copper-transporting ATPase)	• Hepatic: liver disease, hepatomegaly, cirrhosis, acute liver failure • Psychiatric: personality disorders, mood disorders, psychosis, anorexia, sleep disturbances • Neurological: dystonia, dysarthria, dysphagia, dysgraphia, dyslalia, tremor, athetosis, chorea, cerebellar ataxia, cognitive decline	• Kayser-Fleischer rings (gold or gray-brown opacity in the peripheral cornea) • MRI: copper deposits in the brain appearing as hypointense lesions in T_2_-weighted imaging and hyperintense in T_1_-weighted imaging • Labs: total serum Cu↓, urinary Cu↑	• Copper chelators increase urinary copper excretion (penicillamine, trientine) or block the intestinal copper absorption (zinc sulfate) • Liver transplantation
Lysosomal storage disorders	Metabolic leukodystrophies: alpha-L-fucosidosis (*FUCA1*, alpha-L-fucosidase)	• CNS and PNS (in some cases) • Severe neurodegeneration with rapid and progressive mental and motor deterioration • Facial dysmorphia, gingival hypertrophy, angiokeratoma • Visceromegaly • Ocular abnormalities, hearing loss	• MRI: T_2_-weighted hyperintensity in GP reminiscent of early PKAN, generalized hypomyelination of white matter tracks • Labs: urine oligosaccharides and glycoproteins	• No therapeutics approved • Supportive measures and symptom-based interventions
	NCLs, Batten disease (*several genes*)	• Epileptic seizures • Progressive psychomotor decline • Visual failure	• MRI: cerebral and cerebellar atrophy • Blood smear with lymphocyte vacuoles (CLN3)	• CLN2 disease: ERT (cerliponase alpha, Brineura^TM^) • Supportive measures and symptom-based interventions
	NP-C (*NPC1, NPC2*, #257220, #607625)	• Visceral: hepatomegaly • Neurodegenerative: VSGP, cerebellar ataxia, dystonia, dysarthria, dysphagia, seizures, cataplexy • Psychiatric-neurodegenerative: dementia, psychiatric manifestations	• MRI: cerebellar atrophy, thinning of the corpus callosum, cerebral atrophy • Bile acid B • Oxysterols • Filipin test	• Miglustat (Zavesca^TM^, Brazaves^TM^) (in some countries) • Supportive measures and symptom-based interventions
Disorders in pyruvate metabolism	PDH deficiency	• CNS: developmental delay, hypotonia, peripheral neuropathy, pyramidal signs, seizures, Leigh syndrome • Dysmorphic features, microcephaly	• MRI: T_2_-weighted hyperintensity in GP reminiscent of early PKAN • Labs^*^: lactate↑, 2-oxoglutaric acid↑, isoleucine↑, leucine↑, valine↑	• Combined metabolic therapy: • Thiamine supplementation • Emergency treatment during episodes
Organic acidemias and acidurias (OAs)	Glutaric acidemia type 1 (GA-1) (*GCDH*, glutaryl-CoA dehydrogenase)	• Progressive macrocephaly • Encephalopathic crises, acute bilateral striatal injury and complex movement disorders • Insidious-onset basal ganglia injury without a clear acute crisis	• MRI: T_2_-weighted hyperintensity in GP reminiscent of early PKAN • Labs^*^: glutaric acid↑, 3-hydroxyglutaric acid↑, glutarylcarnitine (C5DC) ↑, glutaconic acid↑	• Combined metabolic therapy: • Dietary restriction of lysine • Carnitine supplementation • Emergency treatment during episodes
Inborn errors of purine and pyrimidine (P/P) metabolism	LND (*HPRT*, hypoxanthine-guanine phosphoribosyltransferase)	• Motor dysfunction resembling severe cerebral palsy, choreoathetosis • Intellectual disability • Self-mutilation • Crystalluria, urolithiasis, acute renal failure, gouty arthritis, megaloblastic anemia	• Labs^*^: UA, (hyp-)xan↑	• Treatment aiming at prevention of renal failure from crystal nephropathy and improvement of renal function: • XOR inhibitors (e.g., allopurinol) • High-fluid intake • Alkalization of the urine by administration of bicarbonate or citrate • Low-purine diet
Mitochondrial diseases	Leigh syndrome	• Psychomotor regression • Hypotonia, spasticity, movement disorders, cerebellar ataxia, peripheral neuropathy • Hypertrophic cardiomyopathy	• MRI: T_2_-weighted hyperintensity in GP reminiscent of early PKAN • Labs^*^: blood and CSF lactate↑	• No therapeutics approved • Ketogenic diet • Coenzyme Q

BPAN, beta-propeller protein-associated neurodegeneration; CoPAN, COASY protein-associated neurodegeneration; CNS, central nervous system; Cu, copper; FAHN, fatty acid hydroxylase-associated neurodegeneration; Fe, iron; GP: globus pallidus; LND, Lesch-Nyhan disease; MPAN, mitochondrial membrane protein-associated neurodegeneration; MRI, magnetic resonance imaging; NCLs, neuronal ceroid lipofuscinoses; NPD-C, Niemann Pick Disease Type C; OMIM, Online Mendelian Inheritance in Man; PDH, pyruvate dehydrogenase; PKAN, pantothenate kinase-associated neurodegeneration; PLAN, PLA2G6-associated neurodegeneration; PNS, peripheral nervous system; RN, red nucleus; SN, substantia nigra; UA, uric acid; (hyp-)xan: hypoxanthine; VSGP, vertical supranuclear gaze palsy; XOR, xanthine oxidoreductase.

Eye-of-the-tiger sign = T_2_-weighted hypointense signal in the GP with a central region of hyperintensity.

^*^Refers to high-performance liquid chromatography with UV detection, high performance liquid chromatography-mass spectrometry, gas chromatography-mass spectrometry, electrospray-ionization tandem mass spectrometry or tandem mass spectrometry.

Index metabolites increased (↑) or decreased (↓) in body fluids or in indicated cell types.

## Conclusions

The significant challenges associated with designing a clinical trial in rare disease can sometimes be successfully met through strategic engagement with experts in the rare disease, seeking regulatory and biostatistical guidance, and early involvement of patients and families. The strategic processes used for clinical trial development in the discussed rare disease PKAN can be applied to clinical trial development in other rare diseases, particularly IEMs with movement disorders.

In addition to the discussed strategies, a paradigm shift is needed within the regulatory processes to meet the 21st Century Cures Act and the FDA Action Plan for Rare Neurodegenerative Diseases (68), to help accelerate medical product development and bring new innovations and advances to patients who need them faster and more efficiently, for patients with rare diseases. Regulatory flexibility and support for novel trial designs is needed rather than continuing to require the same design and methodology as for common diseases with large study populations. New ways of looking at novel designs, including within-patient change across a population and more effective use of natural history data when it exists, are needed. Making accelerated approvals of new treatments easier based on proximal disease markers that are not required to go through the entire surrogate biomarker pathway, which is extremely challenging for very rare diseases due to the low numbers of available patients, with subsequent long-term studies, would allow for the treatment of the disease much earlier, or even pre-symptomatically. Early treatment will yield the best long-term QOL outcome for patients receiving the disease-modifying therapy through disease prevention or stabilization of disease at a time when the patient is only minimally affected by the disease process. Without support for such an early intervention and approval process, the drug treatment at a later stage at best might prolong the survival of the last remaining neurons, slow clinical progression, and prolong the time the patient lives in a disabled state. To do better than this, novel trial designs and changes in the approach to rare disease by regulatory authorities will be needed.

## Author contributions

AL, AJ, AV, HP, and JG participated in the original conceptualization and initial draft of the manuscript. AT-S, EB-K, and FE contributed new conceptualizations and substantial revisions of the manuscript. All authors contributed to revisions of the manuscript and approved the submitted manuscript.
